# Transarterial Chemoembolization for Hepatocellular Carcinoma in Clinical Practice: Temporal Trends and Survival Outcomes of an Iterative Treatment

**DOI:** 10.3389/fonc.2022.822507

**Published:** 2022-01-31

**Authors:** Filippo Pelizzaro, Selion Haxhi, Barbara Penzo, Alessandro Vitale, Edoardo G. Giannini, Vito Sansone, Gian Ludovico Rapaccini, Maria Di Marco, Eugenio Caturelli, Donatella Magalotti, Rodolfo Sacco, Ciro Celsa, Claudia Campani, Andrea Mega, Maria Guarino, Antonio Gasbarrini, Gianluca Svegliati-Baroni, Francesco Giuseppe Foschi, Andrea Olivani, Alberto Masotto, Gerardo Nardone, Giovanni Raimondo, Francesco Azzaroli, Gianpaolo Vidili, Maurizia Rossana Brunetto, Franco Trevisani, Fabio Farinati

**Affiliations:** ^1^ Department of Surgery, Oncology and Gastroenterology, Gastroenterology Unit, University of Padova, Padova, Italy; ^2^ Department of Surgery, Oncology and Gastroenterology, Hepatobiliary Surgery and Liver Transplantation Unit, University of Padova, Padova, Italy; ^3^ Gastroenterology Unit, Department of Internal Medicine, University of Genova, Istituto di Ricovero e Cura a Carattere Scientifico (IRCCS) Ospedale Policlinico San Martino, Genova, Italy; ^4^ Division of Internal Medicine, Hepatobiliary and Immunoallergic Diseases, Istituto di Ricovero e Cura a Carattere Scientifico (IRCCS) Azienda Ospedaliero-Universitaria di Bologna, Bologna, Italy; ^5^ Gastroenterology Unit, Fondazione Policlinico Universitario A. Gemelli, Istituto di Ricovero e Cura a Carattere Scientifico (IRCCS), Roma, Italy; ^6^ Medicine Unit, Bolognini Hospital, Seriate, Italy; ^7^ Gastroenterology Unit, Belcolle Hospital, Viterbo, Italy; ^8^ Internal Medicine Unit, Department of Medical and Surgical Sciences, Istituto di Ricovero e Cura a Carattere Scientifico (IRCCS) Azienda Ospedaliero-Universitaria di Bologna, Bologna, Italy; ^9^ Gastroenterology and Digestive Endoscopy Unit, Foggia University Hospital, Foggia, Italy; ^10^ Department of Health Promotion, Mother & Child Care, Internal Medicine & Medical Specialties (PROMISE), Gastroenterology & Hepatology Unit, University of Palermo, Palermo, Italy; ^11^ Department of Surgical, Oncological and Oral Sciences (Di.Chir.On.S.), University of Palermo, Palermo, Italy; ^12^ Department of Experimental and Clinical Medicine, Internal Medicine and Hepatology Unit, University of Firenze, Firenze, Italy; ^13^ Gastroenterology Unit, Bolzano Regional Hospital, Bolzano, Italy; ^14^ Department of Clinical Medicine and Surgery, Gastroenterology Unit, University of Napoli “Federico II”, Napoli, Italy; ^15^ Internal Medicine and Gastroenterology, Fondazione Policlinico Universitario Agostino Gemelli Istituto di Ricovero e Cura a Carattere Scientifico (IRCCS), Università Cattolica del Sacro Cuore, Roma, Italy; ^16^ Gastroenterology Unit, Polytechnic University of Marche, Ancona, Italy; ^17^ Department of Internal Medicine, Ospedale per gli Infermi di Faenza, AUSL Romagna, Faenza, Italy; ^18^ Infectious Diseases and Hepatology Unit, Azienda Ospedaliero-Universitaria di Parma, Parma, Italy; ^19^ Gastroenterology Unit, Ospedale Sacro Cuore Don Calabria, Negrar, Italy; ^20^ Department of Clinical Medicine and Surgery, Hepato-Gastroenterology Unit, University of Napoli “Federico II”, Napoli, Italy; ^21^ Department of Clinical and Experimental Medicine, Clinical and Molecular Hepatology Unit, University of Messina, Messina, Italy; ^22^ Gastroenterology Unit, Department of Surgical and Medical Sciences, Istituto di Ricovero e Cura a Carattere Scientifico (IRCCS) Azienda Ospedaliero-Universitaria di Bologna, Bologna, Italy; ^23^ Department of Medical, Surgical and Experimental Sciences, Clinica Medica Unit, University of Sassari, Azienda Ospedaliero-Universitaria di Sassari, Sassari, Italy; ^24^ Department of Clinical and Experimental Medicine, Hepatology and Liver Physiopathology Laboratory and Internal Medicine Unit, University of Pisa, Pisa, Italy; ^25^ Medical Semeiotics Unit, Department of Medical and Surgical Sciences, Istituto di Ricovero e Cura a Carattere Scientifico (IRCCS) Azienda Ospedaliero-Universitaria di Bologna, Bologna, Italy

**Keywords:** hepatocellular carcinoma, transarterial chemoembolization, survival, iterative treatment, therapeutic hierarchy

## Abstract

**Background:**

Transarterial chemoembolization (TACE) is one of the most frequently applied treatments for hepatocellular carcinoma (HCC) worldwide. In this study, we aimed at evaluating whether and how TACE application and repetition, as well as the related outcome, have changed over the last three decades in Italy.

**Methods:**

Data of 7,184 patients with HCC were retrieved from the Italian Liver Cancer (ITA.LI.CA) database. Patients were divided according to the period of diagnosis in six cohorts: P1 (1988–1993), P2 (1994–1998), P3 (1999–2004), P4 (2005–2009), P5 (2010–2014), and P6 (2015–2019). All the analyses were repeated in the overall patient population and in Barcelona Clinic Liver Cancer (BCLC) B patients, who are the subgroup of HCC patients originally supposed to receive TACE according to guidelines. TACE was defined as either the first or the main (more effective) treatment.

**Results:**

The proportion of patients receiving TACE as first or main therapy declined over time, and less than 50% of BCLC B patients were treated with chemoembolization from P3 onward. Conversely, TACE was widely used even outside the intermediate stage. Survival of TACE-treated patients progressively increased from P1 to P6. Although TACE was performed only once in the majority of patients, there was an increasing proportion of those receiving 2 or ≥3 treatments sessions over time. The overall survival (OS) of patients undergoing repeated treatments was significantly higher compared to those managed with a single TACE (median OS 40.0 vs. 65.0 vs. 71.8 months in 1, 2, and ≥3 TACE groups, respectively; p < 0.0001). However, after a first-line TACE, the adoption of curative therapies provided longer survival than repeating TACE (83.0 vs. 42.0 months; p < 0.0001), which in turn was associated with better outcomes compared to systemic therapies or best supportive care (BSC).

**Conclusions:**

Despite a decline in the percentage of treated patients over time, TACE has still an important role in the management of HCC patients. The survival of TACE-treated patients gradually improved over time, probably due to a better patient selection. Iterative TACE is effective, but an upward shift to curative therapies provides better outcomes while transition to systemic therapies and BSC leads to a worse prognosis.

## Introduction

Liver cancer ranked as the sixth most common cancer and the third leading cause of cancer-related death worldwide in 2020, with approximately 906,000 incident cases and about 830,000 deaths (1). Hepatocellular carcinoma (HCC), which represents about 90% of primary liver cancers, is a leading cause of mortality among cirrhotic patients (2, 3). In most geographical areas, the annual HCC mortality almost equals its incidence, confirming the high mortality rate of this tumor [5-year survival rate of 12%–14% in the United States and 20% in Italy (4, 5)]. Despite efforts to foster surveillance programs, which could allow an earlier diagnosis and increase the percentage of patients amenable to curative treatments (6–8), HCC is frequently detected at an advanced stage, thus precluding the possibility to deliver curative treatments such as liver transplantation (LT), liver resection (LR), or ablation (ABL) (9).

According to the Barcelona Clinic Liver Cancer (BCLC) algorithm, transarterial chemoembolization (TACE) is the standard-of-care treatment in patients with intermediate-stage HCC (9). However, it is also widely used outside the BCLC B stage and this makes TACE one of the most frequently used treatments for HCC in daily clinical practice worldwide (10, 11). TACE is by definition a palliative and iterative treatment, considering the low rates of complete response and the high risk of disease recurrence (12–14). There is no definitive evidence that scheduled TACE at regular intervals (e.g., every 2 months), irrespective of tumor response, has different effects on patient survival than on demand TACE. Nevertheless, the adoption of an aggressive schedule might lead to the development of liver failure in a high proportion of patients, most of whom are also affected by cirrhosis (15). Therefore, this approach has been substantially abandoned, following the recommendation of the guidelines to retreat with TACE only when residual viable tumor is detected at imaging, and to stop performing TACE when 2 subsequent attempts fail to obtain a significant oncologic response (9). Nevertheless, in clinical practice TACE is often repeated several times, particularly in patients with partial response or after recurrence following an initial successful treatment. However, the benefit of retreating with TACE is uncertain, also because survival prediction in these patients is a difficult issue that only complicated recalibration (16) or time-varying models (i.e., mHAP-III) (17) seem to accurately solve. This uncertainty has been increased by the growing availability of several lines of effective systemic therapy based on tyrosine kinase inhibitors, ramucirumab and immunotherapy (18–23). Indeed, systemic therapy may be a valid (and possibly better) alternative to iterative TACE. In order to support the decision to retreat patients, several algorithms, such as ART score (24, 25) and ABCR score (26), have been proposed.

Although TACE is frequently used as treatment of HCC, few studies investigated whether its use has changed over time. Furthermore, little evidence is available regarding the percentage of patients retreated with TACE in real-life clinical practice, the changing trends of this percentage over time, and the outcome of patients retreated with transarterial therapies compared to other therapeutic options. Considering the availability in the Italian Liver Cancer (ITA.LI.CA) database of a large series of patients managed along a period of 30 years, our study aimed to evaluate whether in real-life clinical practice the use of TACE and its outcome have changed over time, as well as the oncologic and clinical characteristics that guide the choice of this treatment. Moreover, we evaluated temporal trends in the attitude to repeat TACE and outcomes of patients managed with iterative treatment sessions.

## Patients and Methods

### Study Groups

In this retrospective study, data were retrieved from the ITA.LI.CA database, a multicenter registry including 7,817 HCC patients consecutively managed from January 1988 to December 2018 in 24 participating Institutions. Data are collected prospectively and updated every 2 years, and their accuracy is controlled by a data manager in the coordinating center (Bologna University).

The management of the ITA.LI.CA database conforms to the Italian legislation on privacy. According to Italian laws, specific patient consent is not mandatory for any retrospective analysis, but patients provided written informed consent for every diagnostic and therapeutic procedure, as well as for having their clinical data anonymously recorded in the database. This study was conducted in accordance with the ethical guidelines of the Declaration of Helsinki, and the study protocol was approved by the Institutional Review Board of the ITA.LI.CA coordinator center (Bologna University; approval number 99/2012/O/Oss).

For the purpose of the present study, all patients with a diagnosis of HCC registered in the ITA.LI.CA database were considered eligible. The only exclusion criterion was the lack of data on variables relevant for the aim of this study, such as tumor stage and treatment. Therefore, from the entire population of patients included in the database (n = 7,817), 633 patients (8.1%) with missing data were excluded (in 153 patients, information on tumor burden or stage was missing, while treatment modality was not recorded in 480 cases), leaving 7,184 patients for the final analysis. These patients were divided in six 5-year cohorts on the basis of the year of diagnosis: P1 (1988–1993), P2 (1994–1998), P3 (1999–2004), P4 (2005–2009), P5 (2010–2014), and P6 (2015–2019). A flowchart of patient selection is provided in **Supplementary Figure 1**.

HCC diagnosis was histologically confirmed in 2,371 patients (33%), whereas in the remaining cases it was based on the radiological criteria (at computed tomography [CT] or magnetic resonance imaging [MRI]), according to guidelines available at the time of diagnosis (9, 27).

In the ITA.LI.CA database, demographic and clinicopathological data, such as age, sex, comorbidities, etiology of the underlying liver disease, main serological parameters [albumin, bilirubin, international normalized ratio (INR), creatinine, platelet count, alpha-fetoprotein (AFP)], Child–Pugh class, Model for End Stage Liver Disease (MELD) score, presence of ascites and hepatic encephalopathy, clinically relevant portal hypertension (CRPH), and Eastern Cooperative Oncology Group performance status (ECOG-PS), are recorded. CRPH diagnosis was based either on unequivocal signs (presence of splenomegaly, varices, ascites) or platelet count <100 × 10^9^/l (28). The database also reports main macroscopic tumor characteristics [location and size, number of nodules, macrovascular invasion (MVI), and extrahepatic spread (EHS)] evaluated with dynamic CT or MRI. In this study, also in order to evaluated the adherence to its therapeutic recommendation, for staging purposes we used the BCLC staging system (9).

The complete sequence of treatments for every patient is also registered in the ITA.LI.CA database. The following treatment groups were considered in the present study: liver transplantation (LT), liver resection (LR), ablative procedures (ABL: percutaneous ethanol injection, percutaneous or laparoscopic thermal ablation), TACE, trans-arterial embolization (TAE), selective internal radiation therapy (SIRT), systemic therapy with sorafenib or other tyrosine kinase inhibitors (SOR), best supportive care (BSC), and other treatments. In all the analyses, we evaluated the first therapeutic choice and the main (i.e., more effective) treatment according to the following hierarchy: LT, LR, ABL, TACE, TAE and SIRT, SOR, and BSC (29). The ITA.LI.CA database reports the treatment modality at each recurrence. In this study, when different rounds of TACE were necessary to achieve a complete treatment (e.g., treatment of lesions in the left lobe and subsequent treatment of nodules in the right lobe), TACE was considered as a single procedure. On the contrary, when repeated at tumor recurrence, TACEs were considered as separate treatments. Regarding technical details, in the ITA.LI.CA database, chemotherapeutic drugs administered as well as the type of TACE (conventional vs. drug-eluting beads) are rarely registered and were not considered in this study. Response to TACE was evaluated using the modified Response Evaluation Criteria in Solid Tumors (mRECIST) and was categorized in complete response (CR), partial response (PR), stable disease (SD), and progressive disease (SD) (30).

### Statistical Analysis

Categorical variables were reported as absolute and relative frequency (percentages), while quantitative variables as median and interquartile range (IQR). Mann–Whitney test was used to compare quantitative variables; meanwhile, χ^2^ test and Fischer’s exact test were used in the comparison of categorical variables as appropriate.

In order to evaluate predictors of TACE treatment compared to potentially radical (LT, LR, and ABL) and palliative (SOR and BSC) treatments, a multinomial logistic regression was performed. Variables significantly or borderline (p ≤ 0.1) associated with treatment category at univariate analysis were included in multivariate models. The multinomial logistic regression analysis was used to establish the variables predicting TACE as first and main treatment in the overall population of patients and in the subgroup of BCLC B patients.

Overall survival (OS), expressed as median and 95% confidence interval (CI), was calculated from diagnosis to death from any cause or last follow-up. For patients alive at the end of the study, survival was censored at December 31, 2018. Survival curves were calculated with the Kaplan–Meier method and compared with the log-rank test. The independent predictors of survival were identified by the multivariate Cox regression analysis, including in the analysis the variables associated with survival (p ≤ 0.1) at the univariate analysis.

In all the analyses, a two-tailed p value <0.05 was considered as significant. Data were analyzed by IBM SPSS Statistics (version 25.0. Armonk, NY: IBM Corp) and GraphPad Prism version 8.3.1 (GraphPad Software, La Jolla, CA, USA).

## Results

### TACE Treatment in the Whole Population

Baseline demographic and clinical characteristics of patients in the six time periods are described in **Table 1**. Compared to P1 patients, those diagnosed in more recent periods were slightly older, were more frequently diagnosed under surveillance, and had less frequently a viral etiology and cirrhosis. More than half of patients in all time periods had CRPH, with slightly lower percentages in P5 and P6. Liver function and AFP levels at diagnosis were similar among subgroups (except for a slightly lower MELD score in P5 and P6, and a lower median AFP level in P6). While the majority of patients presented with a single liver lesion at diagnosis in each time period, tumor size was significantly smaller in P2–P6 as compared to P1. As far as tumor stage at diagnosis is concerned, BCLC B patients progressively decreased, while the proportion of BCLC C patients increased over time.

**Table 1:** Baseline characteristics of the overall population of patients divided according to the period of diagnosis.

Variables	P1 (1988–1993) n=256	P2 (1994–1998) n=370	P3 (1999–2004) n=867	P4 (2005–2009) n=1323	P5 (2010–2014) n=2515	P6 (2015–2019) n=1853
Sex—males	197 (77.0)	279 (75.4)	657 (75.8)	1003 (75.8)	1932 (76.8)	1464 (79.0)
Age (years)	64 (58–68)	64 (57–70)	67 (61–74) *^d^*	68 (60–74) *^d^*	69 (60–75) *^d^*	69 (60–76) *^d^*
Surveillance	126 (49.2)	209 (56.5)	508 (58.6) *^b^*	831 (62.8) *^d^*	1637 (65.1) *^d^*	1073 (57.9) *^a^*
Etiology						
Viral	150 (58.6)	288 (77.8) *^d^*	613 (70.7) *^c^*	832 (62.9)	1443 (57.4)	941 (50.8) *^a^*
Not viral	54 (21.1)	45 (12.2) *^b^*	188 (21.7)	372 (28.1) *^a^*	815 (32.4) *^c^*	712 (38.4) *^d^*
Viral + other	52 (20.3)	37 (10.0) *^c^*	66 (7.6) *^d^*	119 (9.0) *^d^*	257 (10.2) *^d^*	200 (10.8) *^d^*
Liver disease						
Healthy liver	0 (0)	4 (1.1)	8 (0.9)	13 (1.0)	40 (1.6) *^a^*	41 (2.2) *^b^*
NAFLD	0 (0)	0 (0)	3 (0.3)	12 (0.9)	68 (2.7) *^b^*	50 (2.7) *^b^*
Fibrosis	6 (2.3)	7 (1.9)	49 (5.7) *^a^*	48 (3.6)	148 (5.9) *^a^*	139 (7.5) *^b^*
Cirrhosis	250 (97.7)	359 (97.0)	807 (93.1) *^b^*	1250 (94.5) *^a^*	2259 (89.8) *^d^*	1623 (87.6) *^d^*
ECOG-PS						
0	194 (75.8)	216 (58.4) *^d^*	698 (80.5)	923 (69.8)	1740 (69.2) *^a^*	1359 (73.3)
1–2	54 (21.1)	154 (41.6) *^d^*	166 (19.1) *^d^*	344 (26.0) *^d^*	672 (26.7) *^d^*	450 (24.3) *^d^*
3–4	8 (3.1)	0 (0) *^c^*	3 (0.3) *^c^*	56 (4.2)	103 (4.1)	44 (2.4)
CRPH	176 (68.7)	266 (71.9)	567 (65.4)	844 (63.8)	1514 (60.2) *^b^*	1128 (60.9) *^a^*
Child–Pugh						
A	170 (66.4)	234 (63.2)	552 (63.7)	889 (67.2)	1655 (65.8)	1305 (70.4)
B	75 (29.3)	105 (28.4)	256 (29.5)	340 (25.7)	757 (30.1)	465 (25.1)
C	11 (4.3)	31 (8.4)	59 (6.8)	94 (7.1)	103 (4.1)	83 (4.5)
MELD	10 (8–13)	10 (9–13)	10 (8–13)	10 (8–12)	10 (8–12) *^b^*	9 (8–11) *^c^*
AFP (ng/mL)	30.5 (9.0–201.5)	34.0 (9.0–172.8)	23.0 (7.0–210.0)	31.0 (6.0–330.0)	40.0 (5.0–567.0)	12.5 (4.0–239.3) *^d^*
Tumor morphology						
Monofocal	120 (46.9)	182 (49.2)	432 (49.8)	633 (47.8)	1267 (50.4)	990 (53.4)
Multifocal	112 (43.8)	169 (45.7)	375 (43.3)	587 (44.4)	1044 (41.5)	743 (40.1)
Infiltrative	15 (5.8)	15 (4.1)	33 (3.8)	69 (5.2)	141 (5.6)	61 (3.3) *^a^*
Massive	9 (3.5)	4 (1.1) *^a^*	27 (3.1)	34 (2.6)	63 (2.5)	59 (3.2)
Number	1 (1–4)	1 (1–4)	1 (1–3)	1 (1–3)	1 (1–2)	1 (1–2)
Diameter (cm)	3.5 (2.4–5.1)	3.0 (2.2–4.0) *^a^*	3.0 (2.2–4.5) *^a^*	3.0 (2.0–4.5) *^b^*	3.0 (2.0–5.0) *^a^*	3.0 (2.0–4.8) *^c^*
MVI	27 (10.5)	31 (8.4)	110 (12.7)	158 (11.9)	284 (11.3)	206 (11.1)
EHS	0 (0)	2 (0.5)	68 (7.8) *^d^*	139 (10.5) *^d^*	257 (10.2) *^d^*	189 (10.2) *^d^*
BCLC stage						
0	25 (9.8)	29 (7.8)	68 (7.8)	126 (9.5)	261 (10.4)	261 (14.1)
A	107 (41.8)	175 (47.3)	339 (39.1)	459 (34.7) *^a^*	934 (37.1)	685 (37.0)
B	78 (30.5)	101 (27.3)	216 (24.9)	235 (17.8) *^d^*	376 (15.0) *^d^*	264 (14.2) *^d^*
C	34 (13.3)	38 (10.3)	217 (25.0) *^d^*	439 (33.2) *^d^*	856 (34.0) *^d^*	594 (32.1) *^d^*
D	12 (4.7)	27 (7.3)	27 (3.1)	64 (4.8)	88 (3.5)	49 (2.6)
First treatment						
LT	5 (2.0)	16 (4.4)	28 (3.2)	34 (2.6)	49 (2.0)	33 (1.8)
LR	38 (14.8)	40 (10.8)	125 (14.4)	202 (15.3)	418 (16.6)	280 (15.1)
ABL	62 (24.3)	91 (24.6)	306 (35.3) *^c^*	430 (32.5) *^b^*	787 (31.3) *^a^*	608 (32.8) *^b^*
TACE	117 (45.7)	170 (45.9)	245 (28.3) *^d^*	383 (28.9) *^d^*	752 (29.9) *^d^*	528 (28.5) *^d^*
TAE/SIRT	0 (0)	0 (0)	1 (0.1)	3 (0.2)	21 (0.8)	75 (4.0) *^d^*
SOR	0 (0)	0 (0)	0 (0)	53 (4.0) *^c^*	229 (9.1) *^d^*	178 (9.6) *^d^*
BSC	6 (2.3)	7 (1.9)	78 (9.0) *^c^*	146 (11.0) *^d^*	218 (8.7) *^d^*	116 (6.3) *^b^*
Other	28 (10.9)	46 (12.4)	84 (9.7)	72 (5.5) *^b^*	41 (1.6) *^d^*	35 (1.9) *^d^*
Main treatment						
LT	5 (2.0)	22 (6.0) *^a^*	43 (5.0) *^a^*	83 (6.2) *^b^*	121 (4.8) *^a^*	86 (4.6) *^a^*
LR	38 (14.8)	40 (10.8)	127 (14.7)	214 (16.2)	432 (17.2)	306 (16.5)
ABL	62 (24.2)	90 (24.3)	315 (36.3) *^c^*	436 (33.0) *^a^*	862 (34.3) *^b^*	637 (34.4) *^b^*
TACE	117 (45.7)	165 (44.6)	219 (25.3) *^d^*	317 (24.0) *^d^*	597 (23.7) *^b^*	424 (22.9) *^d^*
TAE/SIRT	0 (0)	0 (0)	1 (0.1)	2 (0.2)	18 (0.7)	72 (3.9) *^b^*
SOR	0 (0)	0 (0)	0 (0)	53 (4.0) *^c^*	226 (9.0) *^d^*	176 (9.5) *^d^*
BSC	6 (2.4)	7 (1.9)	78 (9.0) *^c^*	146 (11.0) *^d^*	218 (8.7) *^d^*	116 (6.3) *^b^*
Other	28 (10.9)	46 (12.4)	84 (9.7)	72 (5.4) *^b^*	41 (1.6) *^d^*	36 (1.9) *^d^*

Continuous variables are reported as median and interquartile range (IQR), while categorical variables as absolute and relative frequencies.

The first cohort (1988–1993) is taken as reference in the comparison with other time periods.

a p < 0.05 and ≥0.01.

b p < 0.01 and ≥0.001.

c p < 0.001 and ≥0.0001.

d p < 0.0001.

NAFLD, non-alcoholic fatty liver disease; ECOG-PS, Eastern Cooperative Oncology Group performance status; CRPH, clinically relevant portal hypertension; MELD, Model for End-Stage Liver Disease; AFP, alpha-fetoprotein; MVI, macrovascular invasion; EHS, extrahepatic spread; BCLC, Barcelona Clinic Liver Cancer; LT, liver transplantation; LR, liver resection; ABL, ablation; TACE, transarterial chemoembolization; TAE, transarterial embolization; SIRT, selective internal radiation therapy; SOR, systemic therapy; BSC, best supportive care.

**Figure 1:**
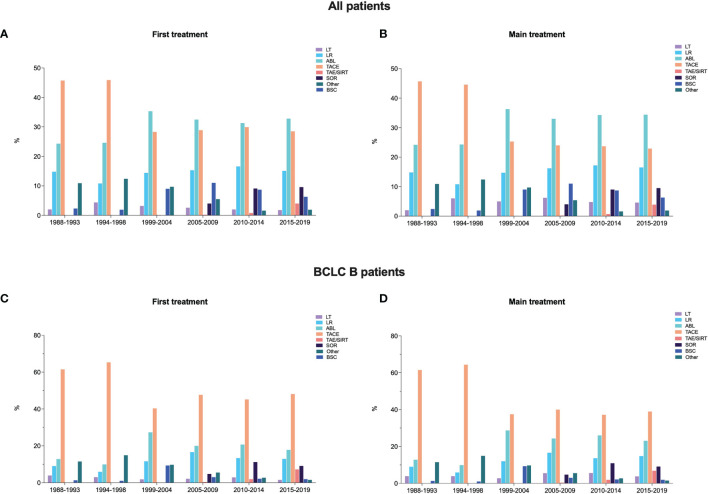
Distribution of the first and main treatment adopted in the overall population of patients **(A, B)** and in BCLC B patients **(C, D)** in the six time periods considered.

The choice of prescribing TACE as the first therapeutic approach decreased across P2 and P3, remaining thereafter substantially stable. Namely, 45.7% of patients in P1, 45.9% in P2, 28.3% in P3, 28.9% in P4, 29.9% in P5, and 28.5% in P6 underwent TACE as first treatment (**Table 1** and **Figure 1A**). A very similar trend was demonstrated for TACE used as the main treatment (45.7%, 44.6%, 25.3%, 24.0%, 23.7%, and 22.9%, respectively) (**Table 1** and **Figure 1B**). In parallel to the decrease in TACE use, there was an increase of ABL and systemic therapies as both first and main treatments. The rate of LT and LR remained approximately stable across the six time periods considered.

### TACE Treatment in BCLC B Patients

Of the entire population of patients included in the study, 1,270 (17.7%) were classified as BCLC B at the time of diagnosis. Baseline demographic and clinical characteristics of these patients in the six time periods considered are shown in **Table 2**. As in the whole population, patients diagnosed in recent time cohorts were slightly older and more frequently diagnosed with HCC under surveillance. Non-viral etiologies increased over time. No statistically significant differences in the percentage of patients with CRPH were demonstrated between different groups. A better residual liver function (as evaluated with Child–Pugh score and MELD) was documented in patients more recently diagnosed. As far as tumor burden is considered, the number of liver lesions was significantly lower in the more recent cohorts while the size of the largest nodule remained stable across the different calendar periods.

**Table 2:** Baseline characteristics of the BCLC B patients divided according to the period of diagnosis.

Variables	P1 (1988–1993) n = 78	P2 (1994–1998) n = 101	P3 (1999–2004) n = 216	P4 (2005–2009) n = 235	P5 (2010–2014) n = 376	P6 (2015–2019) n = 264
Sex—males	68 (87.2)	76 (75.2)	164 (75.9) *^a^*	200 (85.1)	321 (85.4)	231 (87.5)
Age (years)	63 (58–68)	63 (57–70)	67 (60–73) *^b^*	66 (59–72) *^b^*	67 (59–74) *^c^*	68 (59–76) *^c^*
Surveillance	34 (43.6)	54 (53.5)	111 (51.4)	140 (59.6) *^a^*	221 (58.8) *^a^*	124 (47.0)
Etiology						
Viral	44 (56.5)	77 (76.2) *^b^*	151 (69.9) *^a^*	142 (60.4)	206 (54.8)	113 (42.8) *^a^*
Not viral	14 (17.9)	15 (14.9)	44 (20.4)	70 (29.8)	136 (36.2) *^b^*	111 (42.0) *^d^*
Viral + other	20 (25.6)	9 (8.9) *^b^*	21 (9.7) *^b^*	23 (9.8) *^c^*	34 (9.0) *^c^*	40 (15.2) *^a^*
Liver disease						
Healthy liver	0 (0)	3 (3.0)	1 (0.1)	1 (0.4)	13 (3.5)	3 (1.1)
NAFLD	0 (0)	0 (0)	2 (0.9)	3 (1.3)	9 (2.4)	11 (4.2)
Fibrosis	3 (3.8)	1 (1.0)	13 (6.0)	8 (3.4)	25 (6.6)	21 (8.0)
Cirrhosis	75 (96.2)	97 (96.0)	200 (92.6)	223 (94.9)	329 (87.5) *^a^*	229 (86.7) *^a^*
CRPH	51 (65.4)	72 (71.3)	130 (60.2)	131 (55.7)	202 (53.7)	158 (59.8)
Child–Pugh						
A	47 (60.3)	70 (69.3)	144 (66.7)	181 (77.0) *^b^*	283 (75.3) *^a^*	201 (76.1) *^b^*
B	31 (39.7)	31 (30.7)	72 (33.3)	54 (33.0)	93 (24.7)	63 (23.9)
MELD	11 (9-13)	11 (8–13)	10 (9–12)	10 (8–11) *^a^*	10 (8–11) *^c^*	9 (8–11) *^c^*
AFP (ng/mL)	40.0 (13.0-417.0)	30.5 (8.8–272.0)	50.0 (10.5–654.5)	39.5 (8.0–892.5)	92.0 (12.0–1158.0)	47.5 (7.0–1019.0)
Morphology						
2–3 lesions	2 (2.5)	4 (4.0)	35 (16.2) *^b^*	94 (40.0) *^d^*	224 (59.6) *^d^*	166 (62.9) *^d^*
>3 lesions	63 (80.8)	88 (87.1)	158 (73.1)	102 (43.4) *^d^*	111 (29.5) *^d^*	77 (29.2) *^d^*
Infiltrative/massive	13 (16.7)	9 (8.9)	23 (10.6)	39 (16.6)	41 (10.9)	21 (7.9) *^a^*
Number	4 (4-4)	4 (4–4)	4 (4–4) *^a^*	4 (2–4) *^d^*	3 (2–4) *^d^*	3 (2–4) *^d^*
Diameter (cm)	4.5 (3.5-6.7)	4.0 (2.9–5.0) *^a^*	4.0 (3.2–5.9)	4.0 (3.5–5.5)	4.0 (3.6–5.5)	4.0 (3.5–5.8)
First treatment						
LT	3 (3.9)	3 (3.0)	4 (1.8)	5 (2.1)	11 (2.9)	4 (1.5)
LR	7 (9.0)	6 (5.9)	25 (11.6)	39 (16.6)	50 (13.3)	34 (12.9)
ABL	10 (12.8)	10 (9.9)	59 (27.3) *^a^*	47 (20.0)	78 (20.7)	47 (17.8)
TACE	48 (61.5)	66 (65.3)	87 (40.3) *^b^*	112 (47.7) *^a^*	170 (45.2) *^b^*	127 (48.1) *^a^*
TAE/SIRT	0 (0)	0 (0)	0 (0)	1 (0.4)	7 (1.9)	19 (7.2) *^b^*
SOR	0 (0)	0 (0)	0 (0)	11 (4.7)	42 (11.2) *^c^*	24 (9.1) *^b^*
BSC	1 (1.3)	1 (1.0)	20 (9.3) *^a^*	7 (3.0)	8 (2.1)	5 (1.9)
Other	9 (11.5)	15 (14.9)	21 (9.7)	13 (5.5)	10 (2.7) *^b^*	4 (1.5) *^c^*
Main treatment						
LT	3 (3.9)	4 (3.9)	6 (2.8)	13 (5.5)	21 (5.6)	10 (3.8)
LR	7 (9.0)	6 (5.9)	26 (12.0)	39 (16.6)	51 (13.6)	39 (14.8)
ABL	10 (12.8)	10 (9.9)	62 (28.7) *^b^*	57 (24.3) *^a^*	98 (26.0) *^a^*	61 (23.1)
TACE	48 (61.5)	65 (64.4)	81 (37.5) *^c^*	94 (40.0) *^b^*	140 (37.2) *^c^*	103 (39.0) *^c^*
TAE/SIRT	0 (0)	0 (0)	0 (0)	1 (0.4)	7 (1.9)	18 (6.8) *^a^*
SOR	0 (0)	0 (0)	0 (0)	11 (4.7)	41 (10.9) *^c^*	24 (9.1) *^b^*
BSC	1 (1.3)	1 (1.0)	20 (9.3) *^a^*	7 (3.0)	8 (2.1)	5 (1.9)
Other	9 (11.5)	15 (14.9)	21 (9.7)	13 (5.5)	10 (2.7) *^b^*	4 (1.5) *^c^*

Continuous variables are reported as median and interquartile range (IQR), while categorical variables as absolute and relative frequencies.

The first cohort (1988–1993) is taken as reference in the comparison with other time periods.

a p < 0.05 and ≥0.01.

b p < 0.01 and ≥0.001.

c p < 0.001 and ≥0.0001.

d p < 0.0001.

NAFLD, non-alcoholic fatty liver disease; CRPH, clinically relevant porta hypertension; MELD, Model for End-Stage Liver Disease; AFP, alpha-fetoprotein; LT, liver transplantation; LR, liver resection; ABL, ablation; TACE, transarterial chemoembolization; TAE, transarterial embolization; SIRT, selective internal radiation therapy; SOR, systemic therapy; BSC, best supportive care.

As in the whole population, even in BCLC B patients there was a decrease in the use of TACE as the first therapeutic approach between P2 and P3. In fact, 61.5% of patients in P1 and 65.3% in P2 were treated with chemoembolization, while these figures were 40.3% in P3, 47.7% in P4, 45.2% in P5, and 48.1% in P6 (**Table 2** and **Figure 1C**). Despite TACE being the standard of care according to BCLC guidelines, patients with intermediate-stage HCC diagnosed in more recent temporal cohorts underwent TACE as main treatment only in about one third of cases. Indeed, TACE was used as the main treatment in 61.5% of P1, 64.4% of P2, 37.5% of P3, 40.0% of P4, 37.2% of P5, and 39.0% of P6 patients (**Table 2** and **Figure 1D**). Notably, recently diagnosed BCLC B patients more frequently underwent to curative treatments (LR and ABL) as main therapies.

**Figure 2:**
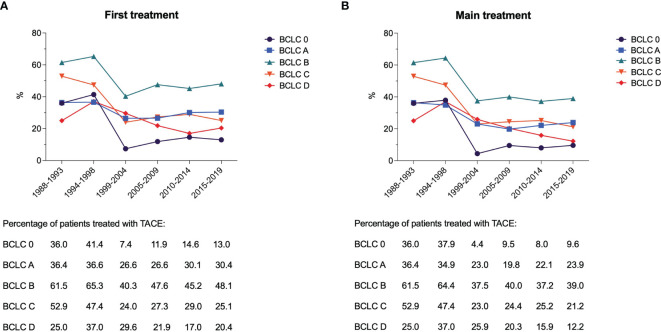
Proportion of patients treated with TACE as first **(A)** and main **(B)** treatment in the six time periods considered, according to the BCLC stage.

Beyond BCLC B patients, TACE was also widely used across all the other HCC stages (**Figure 2**). A substantial subgroup of BCLC 0 and A patients underwent TACE, as both first and main treatments, but even in these cases the use of such treatment dropped over time (from 36.0% in P1 to 9.6% in P6 as main treatment in BCLC 0; from 36.4% in P1 to 23.9% in P6 as main treatment in BCLC A). More than half of BCLC C patients were treated with TACE in P1 (52.9%), while this treatment was used in a lower proportion of patients both as first or main choice (25.1% and 21.2%, respectively) in P6.

### Predictive Factors of Treatment With TACE

The multinomial logistic regression (**Table 3**) showed that, compared to potentially curative options (LT, LR, ABL), TACE was selected preferentially in older patients [adjusted odds ratio (aOR) = 0.88 per 10-year increase, 95% CI 0.82–0.94], in those with non-viral etiology (aOR = 0.82, 95% CI 0.69–0.97), with deteriorated clinical conditions (ECOG-PS ≥1), with CRPH (aOR = 0.51, 95% CI 0.43–0.60), and with poor residual liver function (aOR = 0.96, 95% CI 0.94–0.99, for MELD score). Moreover, patients with high tumor burden (number and size of liver lesions, and AFP levels) were less likely to receive LT/LR/ABL as the first therapeutic option. The same variables, with the addition of EHS (aOR = 0.54, 95% CI 0.36–0.83), were also negatively associated with LT/LR/ABL compared to TACE as main treatment. By contrast, patients with deteriorated clinical conditions (ECOG-PS ≥1), poor liver function, and high tumor burden (number and size of liver tumors, presence of MVI and EHS) were more likely to receive systemic or palliative treatment as compared to TACE, as both first and main therapy. Diagnosis under regular surveillance was significantly associated with higher odds to receive TACE rather than SOR or BSC.

**Table 3:** Multinomial logistic regression showing independent factors associated with probability of receive TACE compared to potentially curative treatment (LT, LR, and ABL) and palliative therapies (SOR and BSC).

Variables		Curative treatment (LT, LR, and ABL)		Palliative treatment (SOR and BSC)		Curative treatment (LT, LR, and ABL)		Palliative treatment (SOR and BSC)	
		First treatment				Main treatment			
		aOR (95% CI)	p	aOR (95% CI)	p	aOR (95% CI)	p	aOR (95% CI)	p
Sex	Females	Ref	–	Ref	–	Ref	–	Ref	–
	Males	0.95 (0.79–1.13)	0.54	0.81 (0.61–1.09)	0.15	1.00 (0.83–1.21)	0.98	0.82 (0.61–1.11)	0.19
Age (per 10-year increase)		0.88 (0.82–0.94)	0.0001	1.08 (0.97–1.21)	0.16	0.81 (0.75–0.87)	<0.0001	1.04 (0.92–1.16)	0.55
Period of diagnosis	P1	Ref	–	Ref	–	Ref	–	Ref	–
	P2	0.11 (0.01–0.88)	0.04	0.57 (0.03–10.75)	0.71	0.20 (0.03–1.37)	0.10	0.62 (0.03–114)	0.75
	P3	0.68 (0.20–2.35)	0.54	2.73 (0.42–17.98)	0.30	0.80 (0.23–2.79)	0.73	2.89 (0.45–18.75)	0.27
	P4	0.61 (0.18–2.08)	0.43	1.96 (0.30–12.69)	0.48	0.81 (0.23–2.79)	0.74	2.21 (0.35–14.13)	0.40
	P5	0.51 (0.15–1.75)	0.29	1.41 (0.22–9.12)	0.72	0.74 (0.22–2.53)	0.63	1.61 (0.25–10.20)	0.62
	P6	0.49 (0.14–1.67)	0.26	1.16 (0.18–7.49)	0.88	0.66 (0.19–2.26)	0.51	1.27 (0.20–8.14)	0.80
Etiology	Viral	Ref	–	Ref	–	Ref	–	Ref	–
	Not viral	0.82 (0.69–0.97)	0.02	1.02 (0.78–1.33)	0.90	0.82 (0.69–0.98)	0.03	1.01 (0.77–1.33)	0.93
	Viral+other	0.90 (0.69–1.17)	0.42	1.31 (0.89–1.94)	0.18	0.87 (0.66–1.14)	0.30	1.31 (0.88–1.96)	0.19
Surveillance	No	Ref	–	Ref	–	Ref	–	Ref	–
	Yes	1.08 (0.91-1.27)	0.38	0.62 (0.48-0.79)	0.0001	1.05 (0.88–1.25)	0.57	0.62 (0.48–0.80)	0.0002
ECOG-PS	0	Ref	–	Ref	–	Ref	–	Ref	–
	1–2	0.65 (0.54–0.78)	<0.0001	2.54 (1.99–3.24)	<0.0001	0.63 (0.53–0.76)	<0.0001	2.46 (1.92–3.16)	<0.0001
	3–4	0.39 (0.17–0.87)	0.02	11.85 (6.25–22.46)	<0.0001	0.35 (0.15–0.77)	0.01	10.71 (5.59–20.55)	<0.0001
CRPH	No	Ref	–	Ref	–	Ref	–	Ref	–
	Yes	0.51 (0.43–0.60)	<0.0001	1.05 (0.80–1.37)	0.75	0.60 (0.51–0.71)	<0.0001	1.11 (0.84–1.46)	0.48
MELD		0.96 (0.94–0.99)	0.001	1.09 (1.06–1.12)	<0.0001	0.96 (0.94–0.98)	0.0002	1.08 (1.05–1.11)	<0.0001
Number		0.66 (0.61–0.70)	<0.0001	1.16 (1.10–1.22)	<0.0001	0.70 (0.65–0.74)	<0.0001	1.14 (1.08–1.21)	<0.0001
Diameter (cm)		0.89 (0.86–0.93)	<0.0001	1.15 (1.10–1.21)	<0.0001	0.88 (0.84–0.92)	<0.0001	1.14 (1.08–1.19)	<0.0001
MVI	No	Ref	–	Ref	–	Ref	–	Ref	–
	Yes	0.80 (0.58–1.11)	0.18	1.75 (1.22–2.49)	0.002	0.73 (0.53–1.01)	0.06	1.61 (1.12–2.31)	0.01
EHS	No	Ref	–	Ref	–	Ref	–	Ref	–
	Yes	0.71 (0.47–1.09)	0.12	4.01 (2.71–5.93)	<0.0001	0.54 (0.36–0.83)	0.004	3.55 (2.40–5.26)	<0.0001
AFP (ng/mL)	≤20	Ref	–	Ref	–	Ref	–	Ref	–
	20–200	0.79 (0.66–0.95)	0.01	0.87 (0.64–1.18)	0.38	0.81 (0.66–0.98)	0.03	0.86 (0.63–1.18)	0.36
	>200	0.61 (0.51–0.74)	<0.0001	1.18 (0.90–1.55)	0.23	0.59 (0.49–0.72)	<0.0001	1.15 (0.87–1.51)	0.34

TACE treatment is the reference category of the multinomial logistic regression. OR < 1 indicates that the variable is associated with higher probability of being treated with TACE rather than the comparison category (potentially curative treatments or palliative treatments). OR > 1 indicates that the variable is associated with higher probability to be treated with potentially curative treatments (or palliative treatments) rather than TACE.

In the multivariate models, BCLC stage was not included in favor of its constituent variables (number of liver tumors, size MVI, EHS, ECOG-PS, and residual liver function). MELD was selected as the variable expressing residual liver function.

LT, liver transplantation; LR, liver resection; ABL, ablation; SOR, systemic therapy; BSC, best supportive care; aOR, adjusted odds ratio; CI, confidence interval; ECOG-PS, Eastern Oncology Group performance status; CRPH, clinically relevant portal hypertension; MELD, Model for End-Stage Liver Disease; MVI, macrovascular invasion; EHS, extrahepatic spread; AFP, alpha-fetoprotein.

The role of residual liver function in the choice of treatment requires further clarification. Compared to TACE, while poor residual liver function was negatively associated with LT, LR, and ABL considered together, this was not the case of patients treated specifically with transplantation. Indeed, higher MELD was a negative predictor of treatment with TACE when compared to LT: with the increase of the MELD score, the probability of being treated with TACE as first (aOR = 0.92, 95% CI 0.87–0.97; p = 0.003) and main treatment (aOR = 0.95, 95% CI 0.91–0.99; p = 0.03) decreased. Poor residual liver function favors LT compared to TACE, but at the same time it might contraindicate LR (particularly when large resections are needed). Therefore, considering the low number of patients managed with LT, it is not surprising that grouping together all curative treatments, the detrimental effect of poor residual liver function on the possibility to treat patients with LR prevailed, and we found that higher MELD was associated with greater probability to receive TACE.

In BCLC B patients, negative independent predictors of potentially curative therapies as first treatment compared to TACE were older age (aOR = 0.78 per 10-year increase, 95% CI 0.65–0.93), presence of CRPH (aOR = 0.44, 95% CI 0.30–0.66), and higher number of liver lesions (aOR = 0.87, 95% CI 0.76–0.99) (**Supplementary Table 1**). As far as the main treatment is concerned, in addition to these variables (age, residual liver function, number of liver nodules), also MELD score (aOR = 0.91, 95% CI 0.85–0.98), size of liver lesions (aOR = 0.91, 95% CI 0.83–0.99), and the period of diagnosis were associated with the probability to receive LT/LR/ABL rather than TACE. Compared to patients diagnosed in P1, those diagnosed from P3 to P6 were more likely to receive potentially curative treatments. Only MELD score (aOR = 1.10, 95% CI 1.01–1.20) and tumor size (aOR = 1.13, 95% CI 1.03–1.23) were independently associated with higher odds of receiving SOR or BSC as first treatment instead of TACE in BCLC B patients. In this subpopulation, tumor diameter was also the only predictive variable independently associated with increased probability of being treated with SOR or BSC as main treatment (aOR = 1.10, 95% CI 1.01–1.21).

### Survival Analysis

**Figure 3:**
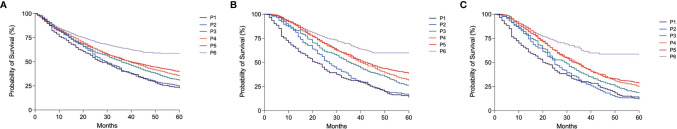
Kaplan–Meier curves showing overall survival according to the period of diagnosis in the overall population of patients **(A)**, in patients treated with TACE as first treatment **(B)** and in those treated with TACE as main treatment **(C)** (all p < 0.0001).

In the whole patient population, the median follow-up was 27.0 months (95% CI 12–54.4), and the median survival was 40.0 months (95% CI 38.4–41.6). The median survival of patients gradually improved from 28.0 months (95% CI 23.2–32.8) in P1 to 40.0 months (95% CI 36.9–43.1) in P5 and it was not evaluable in P6 (p < 0.0001) (**Figure 3A**).

Similar trends were observed in patients treated with TACE as initial treatment (median OS 21.0 months [95% CI 16.2–25.8] in P1, 42.0 months [95% CI 37.7–46.3] in P5 and not estimable in P6; p < 0.0001) (**Figure 3B**). Median OS was generally lower in patients treated with TACE as main therapy, but the improvement of prognosis over time was confirmed in this subgroup (**Figure 3C**). After adjustment for confounders (age, etiology, surveillance, CRPH, MELD, AFP level, BCLC stage, and treatment, this latter only in the whole patient population), the improvement of survival over time was confirmed in all patients and in those treated with TACE as both first and main treatments (**Table 4**).

**Table 4:** Survival analysis according to the period of diagnosis in the overall population of patients.

Period of diagnosis	Median OS (months)	5-year survival (%)	aHR (95% CI)^a^	p
All patients				
P1	28.0 (23.2–32.8)	22.9	Ref	–
P2	28.0 (23.2–32.8)	24.2	1.00 (0.83–1.21)	0.99
P3	36.0 (32.6–39.4)	30.8	0.83 (0.71–0.98)	0.03
P4	39.9 (36.5–43.4)	35.4	0.67 (0.57–0.79)	<0.0001
P5	40.0 (36.9–43.1)	39.9	0.61 (0.52–0.71)	<0.0001
P6	NE (NE-NE)	58.5	0.49 (0.41–0.58)	<0.0001
Patients treated with TACE as first therapy				
P1	21.0 (16.2–25.8)	13.9	Ref	–
P2	27.0 (23.6–30.4)	16.6	0.96 (0.74–1.24)	0.74
P3	36.0 (31.4–40.6)	26.4	0.60 (0.46–0.77)	<0.0001
P4	40.0 (35.6–44.4)	31.6	0.51 (0.40–0.65)	<0.0001
P5	42.0 (37.7–46.3)	38.9	0.45 (0.36–0.57)	<0.0001
P6	NE (NE-NE)	59.7	0.31 (0.24–0.40)	<0.0001
Patients treated with TACE as main therapy				
P1	20.0 (15.0–25.0)	12.5	Ref	–
P2	25.0 (21.7–28.3)	11.9	0.97 (0.75–1.26)	0.84
P3	29.0 (23.8–34.1)	18.8	0.70 (0.54–0.90)	0.006
P4	34.0 (29.7–38.3)	24.8	0.61 (0.47–0.77)	<0.0001
P5	33.0 (29.3–36.6)	28.6	0.57 (0.45–0.73)	<0.0001
P6	NE (NE-NE)	58.6	0.38 (0.29–0.50)	<0.0001

^a^Adjusted for: age, etiology, surveillance, CRPH, MELD, AFP level, BCLC stage, and main treatment (this latter only in the group including all patients).

OS, overall survival; aHR, adjusted hazard ratio; NE, not estimable; TACE, transarterial chemoembolization.

**Figure 4:**
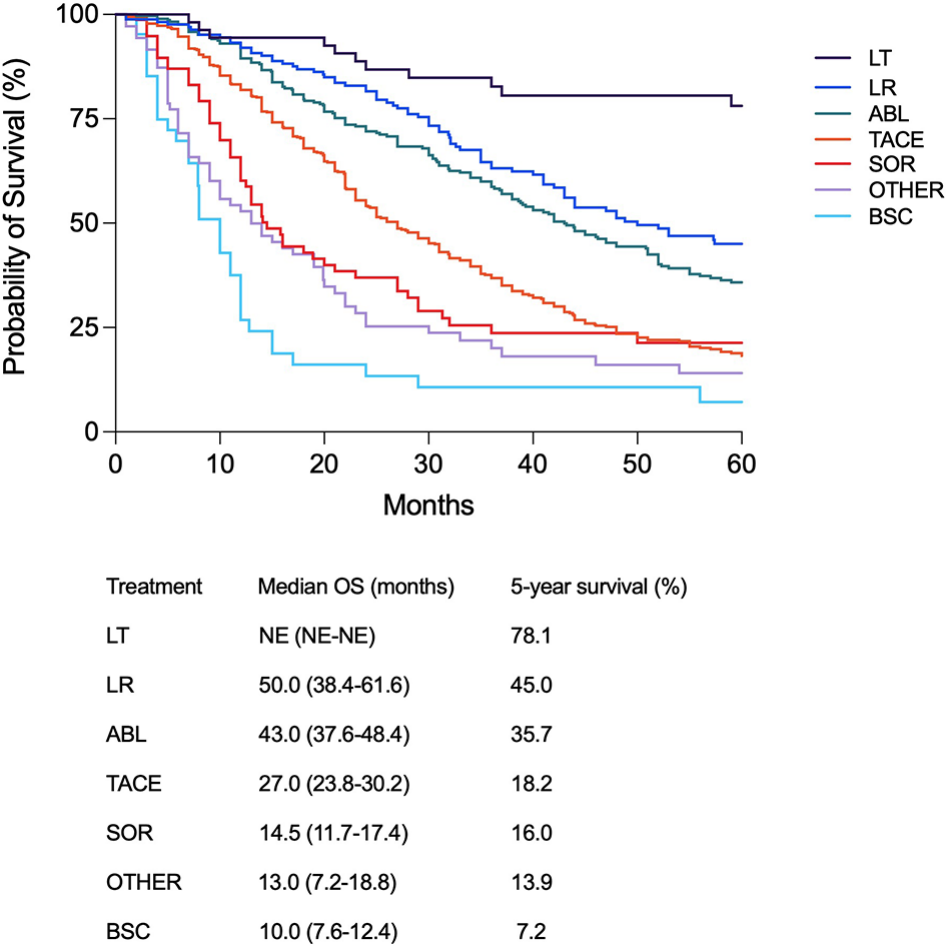
Kaplan–Meier curves showing survival according to the main treatment modality in BCLC B patients (p < 0.0001). Median overall survival and 5-year survival rate are also shown for each treatment modality.

In BCLC B patients, the median follow-up was 24.0 months (95% CI 23.0–26.0) and the median OS was 32.0 months (95% CI 29.5–34.5). The median OS improved over time, from 16.0 months (95% CI 12.2–19.8) in P1 to 35.0 months (95% CI 30.0–40.0) in P5 and not estimable in P6 (p < 0.0001) (**Supplementary Figure 2A**). This gradual OS improvement was confirmed in intermediate-stage patients treated with TACE as both first (**Supplementary Figure 2B**) and main therapies (**Supplementary Figure 2C**). Similar to the results achieved in the whole patient population, the over time improvement of survival was confirmed after correction for confounders (**Supplementary Table 2**). Interestingly, in BCLC B patients a therapeutic hierarchy in terms of survival benefit (LT, LR, ABL, TACE, SOR, BSC) was demonstrated. Longer survival was shown in patients managed with potentially curative treatments compared to TACE which, in turn, was able to improve OS compared to systemic therapies (**Figure 4**). The independent prognostic role of treatment, with an established therapeutic hierarchy, was confirmed in BCLC B patients after adjustment for confounders (results of the Cox multivariate analysis are shown in **Supplementary Table 3**).

### Temporal Trends and Survival of Patients Repeatedly Treated With TACE

**Figure 5:**
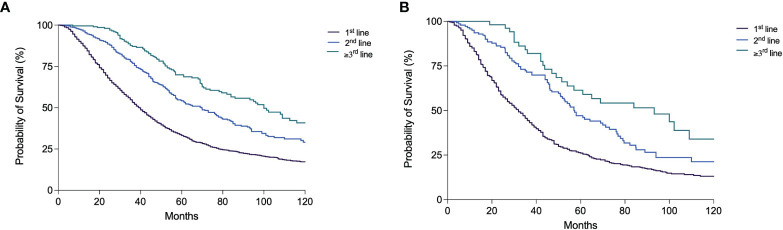
Kaplan–Meier curves showing overall survival according to the line (1st, 2nd, ≥3rd) of TACE treatment during the patient clinical history in the overall patient population **(A)** and in BCLC B patients **(B)** (both p < 0.0001).

Three thousand and seven patients (41.9%) underwent at least a TACE in their clinical history, irrespective of the treatment sequence adopted. The percentage of these patients remained substantially stable across the calendar periods considered, except for P6 in which a lower proportion of patients who received this treatment was registered (35.4%). In BCLC B patients, these percentages were higher compared to the overall population in all the time periods; P3 was the cohort with the lower number of TACE-treated patients (65.3%), while in P4 the highest proportion was registered (91.1%) (**Table 5**). Both in the whole patient population and in the BCLC B group, a forward shift of TACE treatment in the therapeutic sequence was observed over time. Indeed, the proportion of TACE applied as first-line treatment decreased, and consequently its adoption in second and subsequent lines increased (**Table 5** and **Supplementary Figure 3**). Treatment with TACE at recurrence (in second or subsequent lines), after the adoption of hierarchically superior treatments, was associated with better prognosis (**Figure 5**).

**Table 5:** Characteristics of TACE treatment in the different calendar periods.

	P1	P2	P3	P4	P5	P6
All patients						
Patients with at least a TACE	123/256 (48.0)	195/370 (52.7)	354/867 (40.8)^a^	601/1323 (45.4)	1078/2515 (42.9)	656/1853 (35.4) *^c^*
Line of TACE treatment						
1st line	117 (95.1)	170 (87.2) *^a^*	245 (69.2) *^d^*	383 (63.7) *^d^*	752 (69.7) *^d^*	528 (80.5) *^d^*
2nd line	6 (4.9)	17 (8.7)	61 (17.2) *^c^*	143 (23.8) *^d^*	237 (22.0) *^d^*	102 (15.5) *^c^*
≥3rd line	0 (0)	8 (4.1) *^a^*	48 (13.6) *^d^*	75 (12.5) *^d^*	89 (8.3) *^d^*	26 (4.0) *^a^*
Rounds of TACE per patient						
1	123 (100.0)	194 (99.9)	325 (91.8) *^c^*	431 (71.7) *^d^*	631 (58.6) *^d^*	446 (68.0) *^d^*
2	0 (0)	0 (0)	9 (2.5)	102 (17.0) *^d^*	257 (23.8) *^d^*	141 (21.5) *^d^*
≥3	0 (0)	1 (0.1)	20 (5.7) *^b^*	68 (11.3) *^d^*	190 (17.6) *^d^*	69 (10.5) *^d^*
Response to first TACE						
CR + PR	96 (78.1)	164 (84.1)	274 (77.4)	475 (79.0)	863 (80.1)	529 (80.7)
SD + PD	27 (21.9)	31 (15.9)	80 (22.6)	126 (21.0)	215 (19.9)	127 (19.3)
TACE as main treatment	117/123 (95.1)	165/195 (84.6) *^b^*	219/354 (61.9) *^d^*	317/601 (52.7) *^d^*	597/1078 (55.4) *^d^*	424/656 (64.6) *^d^*
BCLC B patients						
Patients with at least a TACE	61/78 (78.2)	82/101 (81.2)	141/216 (65.3) *^a^*	214/235 (91.1) *^b^*	329/376 (87.5) *^a^*	204/264 (77.3)
Line of TACE treatment						
1st line	48 (78.7)	66 (80.5)	87 (61.7) *^a^*	112 (52.3) *^c^*	170 (51.7) *^d^*	127 (62.3) *^a^*
2nd line	13 (21.3)	16 (19.5)	32 (22.7)	70 (32.7)	123 (37.4) *^a^*	56 (27.4)
≥3rd line	0	0	22 (15.6) *^c^*	32 (15.0) *^c^*	36 (10.9) *^b^*	21 (10.3) *^b^*
Rounds of TACE per patient						
1	61 (100.0)	82 (100.0)	134 (95.0)	156 (72.9) *^d^*	195 (59.3) *^d^*	131 (64.2) *^d^*
2	0 (0)	0 (0)	2 (1.4)	32 (15.0) *^c^*	75 (22.8) *^d^*	51 (25.0) *^d^*
≥3	0 (0)	0 (0)	5 (3.6)	26 (12.1) *^b^*	59 (17.9) *^d^*	22 (10.8) *^b^*
Response to first TACE						
CR + PR	45 (73.8)	65 (79.3)	108 (76.6)	163 (76.2)	234 (71.1)	157 (77.0)
SD + PD	16 (26.2)	17 (20.7)	33 (23.4)	51 (23.8)	95 (29.9)	47 (23.0)
TACE as main treatment	48/61 (70.7)	65/82 (79.3)	81/141 (57.4) *^b^*	94/214 (43.9) *^d^*	140/329 (42.6) *^d^*	103/204 (50.5) *^c^*

All patients receiving at least a TACE, irrespective of the treatment sequence adopted, were considered.

The first cohort (P1, 1988–1993) is taken as reference in the comparison with other time periods.

Continuous variables are reported as median and interquartile range (IQR), while categorical variables as absolute and relative frequencies.

a p < 0.05 and ≥0.01.

b p < 0.01 and ≥0.001.

c p < 0.001 and ≥0.0001.

d p < 0.0001.

TACE, trans-arterial chemoembolization; CR, complete response; OR, partial response; SD, stable disease; PD, progressive disease; BCLC, Barcelona Clinic Liver Cancer.

The objective response (CR+PR) to the first TACE was 79.8% in the whole population and 74.9% in BCLC B patients. No significant differences were demonstrated in radiological response, both overall and in BCLC B patients. In the whole population, patients with objective response had a longer median OS compared to non-responders [61.0 months (95% CI 56.0–66.0) vs. 41.0 months (95% CI 34.3–47.7); p < 0.0001) (**Supplementary Figure 4A**). A statistically significant difference in survival between responders [46.2 months (95% CI 40.9–51.5)] and non-responders [32.1 months (95% CI 21.2–43.0)] was also demonstrated in BCLC B patients (p = 0.004) (**Supplementary Figure 4B**).

**Figure 6:**
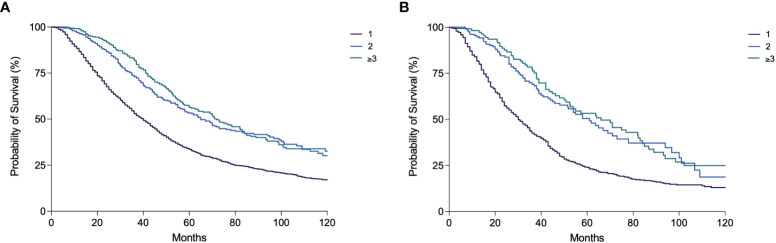
Kaplan–Meier curves showing overall survival according to the number of TACE performed in the overall patient population **(A)** and in BCLC B patients **(B)** (both p < 0.0001).

While in P1–P3 periods the vast majority of patients received only one session of TACE (91.8%–100.0%), in P4–P6 periods a significantly higher percentage of patients received ≥2 TACEs. An increase over time of the percentage of patients treated with several TACE sessions was also observed in BCLC B patients (**Table 5**). Nevertheless, in all calendar periods, both overall and in the intermediate stage, the percentage of patients treated with only 1 TACE was above 50%. The median OS of patients receiving only one TACE [40.0 months (95% CI 37.7–42.3)] was significantly lower compared to patients receiving 2 [65.0 months (95% CI 57.1–72.9)] and 3 or more TACE sessions [71.8 months (95% CI 61.1–82.4)] (p < 0.0001) (**Figure 6A**). In BCLC B patients, comparable results were obtained [30.4 months (95% CI 27.4–33.4) vs. 61.0 (95% CI 49.3–72.7) vs. 66.0 (95% CI 47.0–85.0), respectively; p < 0.0001) (**Figure 6B**). Among the patients who received at least one TACE, 1,805 (60.0%) were dead at the end of the follow-up, mainly because of tumor progression (66.2%) and less frequently from liver decompensation (20.1%) or other causes (13.7%). The proportion of deaths from liver decompensation in patients treated with two (20.4%) and three or more TACEs (18.4%) was similar to that of patients receiving only one course of TACE (20.3%). The majority of patients in the three groups died from tumor progression (67.3% in the 1 TACE group, 61.3% in the 2 TACE group, and 65.8% in the ≥3 TACE group).

**Figure 7:**
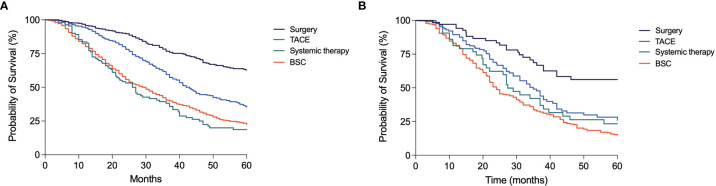
Kaplan–Meier curves showing the survival of patients treated with TACE in first-line according to the subsequent treatment. **(A)** In the overall patient population, those allocated to surgery had a significantly longer OS compared to those receiving another TACE (p < 0.0001); these latter patients had in turn a better prognosis compared to those allocated to systemic therapies (p < 0.0001) or BSC (p < 0.0001). **(B)** In BCLC B patients, those treated with surgery had a better prognosis compared to patients repeating a second course of TACE (p = 0.002); these latter had a similar survival compared to patients treated with systemic therapies (p = 0.44) but maintained a significantly longer survival compared to those allocated to BSC (p = 0.001).

In assessing whether TACE repetition can be considered as a positive or negative approach to the HCC treatment, the OS of patients who underwent an additional TACE in case of non-response or at the time of recurrence was compared to that of patients subsequently treated by curative treatments (LT, LR, or ABL), with an upward shift, or by systemic treatments and BSC, with a downward transition. The upward shift after a TACE was associated with a significantly better survival compared to TACE repetition [83.0 months (95% CI 64.3–101.8) vs. 42.0 months (95% CI 38.4–45.7); p < 0.0001]. This latter, in turn, provided a survival advantage compared to systemic therapies [27.0 months (95% CI 22.3–31.7); p < 0.0001] or BSC [29.0 months (95% CI 26.6–31.4); p < 0.0001] (**Figure 7A**). Similarly, in BCLC B patients, the upward shift after TACE led to a longer survival compared to a second TACE session [69.0 months (95% CI 29.7–108.3) vs. 35.0 months (95% CI 29.6–40.4); p = 0.002]. Instead, the prognosis was similar in patients repeating TACE and in those receiving systemic therapies [27.4 months (95% CI 22.3–32.5); p = 0.44], while patients allocated to BSC had a significantly poorer prognosis [24.0 months (95% CI 21.9–26.1]; p = 0.001) (**Figure 7B**].

## Discussion

With the single exception of LT, in most instances a single treatment, all therapies used in patients with HCC can be considered as iterative. In fact, the risk of tumor recurrence is high even after curative treatments (31), and both LR and ABL have been demonstrated to be safe and effective when repeated (32–37). Also, systemic therapy can be seen as iterative, since drugs for first-, second-, and even third-line therapy are now available (18–23). TACE, one of the most frequently used therapeutic strategies worldwide (10), could be considered by definition an iterative treatment, based on the low rates of complete response achievable and the high recurrence risk with this approach (12–14). Local tumor progression can generally benefit from repeated TACE sessions, but subsequent intra-arterial treatments have been indicated as responsible for an impairment of liver function (15). Although the evidence of TACE effectiveness for HCC treatment dates back of about 20 years (12, 38), there is a lack of studies exploring whether and how the application of TACE and its relative survival benefit changed over time in real-life clinical scenarios. Moreover, even less is known on TACE when considered as an iterative treatment, with few data available regarding the proportion of patients undergoing repetitive sessions. In order to give an answer to these questions, we analyzed the ITA.LI.CA database, one of the largest registries in Europe collecting data of HCC patients managed in many referral Italian centers over more than three decades.

The results of this study indicate that, although declining over time, the percentage of patients treated with TACE remained rather elevated in all the calendar periods considered. TACE was indeed selected as first-line therapeutic choice in 45.7% of patients diagnosed in P1, and the percentage of these cases decreased from P3 onward, until a figure of 28.5% in the last cohort (P6). The same trend was demonstrated when TACE was considered as the main (most radical) treatment applied, and less than a quarter of patients underwent TACE in P4–P6. Similar trends were detected in BCLC B patients, for whom TACE is considered as the standard-of-care treatment according to the BCLC algorithm (9). Although a decline in its application as both first and main therapy was shown, the proportion of patients treated with TACE has stabilized in the last temporal cohorts and it is unlikely to decline further, as it remains a well-established option in the therapeutic algorithm of patients with HCC.

A not negligible proportion of patients in BCLC A, C, and D stages was treated with TACE, and also some very-early stage patients received this treatment. Similarly to the trend demonstrated in the overall patient population and in BCLC B, in the other stages the percentage of patients receiving TACE was higher in P1 and P2 and gradually decreased thereafter. These results show that, in our country, the real-life therapeutic management of HCC frequently deviates from the therapeutic recommendations of the BCLC algorithm. A study investigating the management of HCC in the Campania region of Italy (39), as well as numerous studies worldwide (10, 40–45), obtained comparable results regarding the poor adherence to guidelines, especially in intermediate and advanced stages. Indeed, adhering to BCLC therapeutic recommendations has been questioned by the vast amount of evidence demonstrating the better outcomes of patients undergoing treatments with potentially higher efficiency compared to the BCLC standard of care, and showing that the treatment is an independent predictor of survival within each BCLC stage (28, 42–48). Pertinently, a hierarchy of treatments in terms of survival benefit has been recently demonstrated in each tumor stage (29, 49). Treatment selection in patients with HCC is a difficult issue, and several variables have to be considered. They include not only tumor burden, residual liver function, and clinical conditions but also location of the tumor in the liver, presence of significant portal hypertension, comorbidities, patient preference, and, most importantly, the expected survival benefit of different treatment modalities. All of these are pivotal parameters that must be considered in order to tailor the treatment to the patient, with the aim of maximizing survival outcomes (49).

Despite being TACE the prototype of iterative treatments, our results demonstrated that in the “real life” of the ITA.LI.CA centers most patients (both overall and in BCLC B stage) are treated with TACE only once during their clinical history. In the most recent cohorts compared to the previous ones, a greater proportion of patients were treated with 2 or ≥3 sessions of TACE, but patients who repeated the treatment remained a minority. Considering the attitude to repeat the treatment according to response, presumably patients undergoing several sessions of TACE were those with good tumor responses and a delayed recurrence or a slow progression of the treated lesion(s). Indeed, the survival of patients managed with 2 and ≥3 TACE during their clinical history was significantly longer than that of patients treated with a single TACE course. Moreover, although immortal-time bias may have played a role, this result probably reflects also the better prognosis of those patients who can be retreated at recurrence thank to favorable oncologic and clinical characteristics. Interestingly, repeating TACE did not seem to be associated with an increased risk of death from liver decompensation, since the proportion of patients who died from liver failure was similar in those receiving 1, 2, or ≥3 treatment sessions. However, this comforting finding could not be reproduced if HCC patients are managed outside expert centers.

Although repeating TACE in clinical practice was effective and safe, we also demonstrated that, whenever possible, potentially curative treatments should be preferred to TACE repetition in case of non-response or at the time of cancer recurrence after the first transarterial treatment. In fact, regardless of the tumor stage as well as in BCLC B patients, the upward shift toward curative therapies (LT, LR, and ABL) made possible by TACE provided a longer survival compared to TACE repetition. The latter, in turn, was associated with better prognosis compared to systemic treatment or BSC. Since the survival of HCC patients is largely determined by the more effective treatment received, irrespective of the therapeutic sequence adopted (29), it was not surprising that, after a first-line TACE, the adoption of treatment that can provide a higher survival benefit was associated with better prognosis. Moreover, it has already been demonstrated that surgical treatment of HCC recurrence is a favorable prognostic factor (41, 50, 51). Therefore, the principle of firstly considering the therapy with the highest survival benefit is also valid in the second-line setting, in case of non-response or recurrence after the frontline therapy (49).

As expected, the variables impacting in treatment selection pertained to clinical conditions, residual liver function, and tumor burden. TACE was preferred to curative approaches in older patients, in those with ECOG-PS≥1, CRPH, higher MELD (except for LT specifically), and greater tumor burden (in terms of number and size of nodules, MVI, EHS, and high AFP levels). The opposite was found comparing TACE vs. more palliative treatments: patients who had compromised clinical conditions, higher MELD, increasing number and size of liver nodules, and presence of MVI or EHS were more likely to receive SOR or BSC. In BCLC B patients, age, CRPH, residual liver function, and number and size of liver nodules influenced the selection of treatment. However, the probability of being treated with potentially curative therapies instead of TACE as main treatment increased from P3 onward, suggesting that the attitude of treating intermediate-stage patients with curative intent, whenever feasible, has progressively gained field in recent years.

Another key finding of this study is the progressive improvement of survival over time, not only irrespective of treatment, but also in patients treated with TACE as first-line or main therapy. This improvement occurred also in BCLC B patients, even if the median OS registered were lower in this group. In general, the progressive prolongation of survival may be the result of an earlier HCC diagnosis, a better management and the availability of effective therapies for the underlying liver disease (52), and a better HCC management. In patients treated with TACE, a better selection of patients and technical advancements [e.g., superselective embolization to minimize ischemic injury to non-tumor tissue (53)] are probably the key determinants. In support to these considerations, it has already been demonstrated that refinements in the selection criteria, made possible by the publication of studies demonstrating TACE efficacy in selected patients, provided better survival outcomes despite the more advanced tumor stage of treated patients (54).

Despite this improvement, in intermediate-stage patients, TACE remained less effective in terms of survival benefit than curative treatments. As already reported (48), TACE provided worse outcomes compared to LT, LR, and ABL. Moreover, as the existence of a therapeutic hierarchy in BCLC B patients (LT > LR > ABL > TACE > SOR > BSC) was confirmed by our study, such evidence reinforces the concept that, whenever possible and once having excluded specific contraindications, the treatment potentially offering the best survival should be chosen irrespective of the stage (29, 49).

Despite its many strengths, our study also has some limitations, the most important of which is its retrospective nature which may have introduced unintended biases. Nevertheless, the aim of the study itself, which was to evaluate if and how the application of TACE and the attitude to repeat this treatment in clinical practice have changed in the last decades, required the analysis of a large dataset collecting real-life data. The ITA.LI.CA database offered us this opportunity, having collected data of HCC patients managed in clinical practice for more than three decades and being nowadays one of the largest European databases. However, the retrospective design of the study made it impossible to determine the exact reasons behind the choice of TACE as the first-line or main HCC treatment. Moreover, the reasons that prompted clinicians to prescribe additional TACE after a first session or to switch to other treatments were not predefined and standardized among centers. We tried to evaluate which factors were associated with a higher likelihood of receiving TACE compared to other treatments, but we could not consider all the variables implicated, including patients’ unwillingness to accept the treatment, comorbidities, and technical contraindications. Another major limitation of this study is that we could not provide technical details about TACE treatment. This therapy, which can be grossly divided in conventional TACE (cTACE) and TACE with drug-eluting beads (DEB-TACE), lack in standardization and is a rather heterogeneous treatment (11). Unfortunately, in the ITA.LI.CA database a detailed description of the type of TACE is seldom available and therefore we could not assess the technical evolution of the procedure over time (which may partly explain the progressively better survival seen in recent years) and whether the attitude to treat patients with cTACE or DEB-TACE has changed. Technical skills and experience are fundamental for the effectiveness of TACE. Even though we did not measure these variables, all the Institutions collaborating to the ITA.LI.CA project are expert centers in the management of HCC patients that routinely performs TACE.

In conclusion, in this study we provided a comprehensive analysis of the changes in TACE treatment that have occurred in real-life clinical practice over the last three decades. The proportion of patients treated with TACE, also when BCLC B patients were specifically considered, declined over time but remained stable over the last calendar periods considered. In the real-world clinical management of HCC, a substantial proportion of BCLC B patients are managed deviating from treatment recommendations of Western guidelines, and a relevant percentage of patients belonging to other stages are treated with TACE, confirming that expert centers have a poor adherence to BCLC indications. The better selection of patients, as well as the procedural improvements, may explain the progressive better survival observed over time in patients undergoing TACE. Nevertheless, although this treatment could be safely and effectively repeated in expert centers, in this setting the majority of patients are treated with TACE only once during their clinical history. After a first-line TACE, a shift toward curative therapies (LT, LR, and ABL) to refine the achieved result provides a higher survival benefit compared to TACE repetition and, therefore, it should be preferred whenever feasible.

## Data Availability Statement

The original contributions presented in the study are included in the article/**Supplementary Material**. Further inquiries can be directed to the corresponding author.

## Ethics Statement

The studies involving human participants were reviewed and approved by the Independent Ethic Committee of S. Orsola-Malpighi hospital of Bologna. Written informed consent for participation was not required for this study in accordance with the national legislation and the institutional requirements.

## Other members of the Italian Liver Cancer (ITA.LI.CA) group

*Department of Medical and Surgical Sciences, Semeiotics Unit, University of Bologna, Bologna*: Maurizio Biselli, Paolo Caraceni, Lorenzo Lani, Annagiulia Gramenzi, Davide Rampoldi, Nicola Reggidori, Valentina Santi, Benedetta Stefanini.

*Division of Internal Medicine, Hepatobiliary and Immunoallergic Diseases, IRCCS Azienda Ospedaliero-Universitaria di Bologna, Bologna*: Fabio Piscaglia, Francesco Tovoli, Alessandro Granito, Matteo Tonnini, Alma Di Carlo.

*Department of Surgical and Medical Sciences, Gastroenterology Unit, Alma Mater Studiorum–University of Bologna, Bologna*: Elton Dajti, Giovanni Marasco, Federico Ravaioli.

*Department of Specialist, Diagnostic and Experimental Medicine, Radiology Unit, University of Bologna, Bologna*: Alberta Cappelli, Rita Golfieri, Cristina Mosconi, Matteo Renzulli.

*Department of Surgery, Oncology and Gastroenterology, Gastroenterology Unit, University of Padova, Padova*: Barbara Penzo, Elisa Pinto, Giorgio Palano, Federica Bertellini.

*Gastroenterology and Digestive Endoscopy Unit, Foggia University Hospital, Foggia*: Ester Marina Cela, Antonio Facciorusso.

*Gastroenterology Unit, Department of Internal Medicine, University of Genova, IRCCS Ospedale Policlinico San Martino, Genova, Italy*: Giulia Pieri, Maria Corina Plaz Torres, Francesco Calabrese, Shirin Djahandideh.

*Internal Medicine and Gastroenterology, Fondazione Policlinico Universitario Agostino Gemelli IRCCS, Università Cattolica del Sacro Cuore, Roma*: Nicoletta de Matthaeis, Francesca Romana Ponziani.

*Liver Injury and Transplant Unit, Polytechnic University of Marche, Ancona*: Gloria Allegrini.

*Gastroenterology Unit, Belcolle Hospital, Viterbo*: Giorgia Ghittoni, Valentina Lauria, Giorgio Pelecca.

*Medicina Protetta - Infectious Diseases Unit, Belcolle Hospital, Viterbo*: Serena Dell’Isola.

*Vascular and Interventional Radiology Unit, Belcolle Hospital, Viterbo*: Fabrizio Chegai, Fabio Coratella, Mariano Ortenzi.

*Infectious Diseases and Hepatology Unit, Azienda Ospedaliero-Universitaria of Parma, Parma*: Gabriele Missale, Elisabetta Biasini.

*Gastroenterology Unit, IRCCS Sacro Cuore Don Calabria Hospital, Negrar*: Alessandro Inno, Fabiana Marchetti.

*Department of Health Promotion, Mother & Child Care, Internal Medicine & Medical Specialties, PROMISE, Gastroenterology & Hepatology Unit, University of Palermo, Palermo*: Giuseppe Cabibbo, Calogero Cammà, Paolo Giuffrida, Caterina Stornello, Mauro Grova, Carmelo Marco Giacchetto, Gabriele Rancatore, Maria Vittoria Grassini.

*Department of Clinical and Experimental Medicine, Clinical and Molecular Hepatology Unit, University of Messina, Messina*: Maria Stella Franzè, Carlo Saitta.

*Department of Medical, Surgical and Experimental Sciences, Azienda Ospedaliero-Universitaria of Sassari, Sassari*: Assunta Sauchella.

*Department of Internal Medicine, Ospedale per gli Infermi di Faenza, AUSL Romagna, Faenza*: Vittoria Bevilacqua, Alberto Borghi, Marco Domenicali, Fabio Conti, Emanuela Giampalma, Lucia Napoli, Alessandro Mussetto.

*Department of Experimental and Clinical Medicine, Internal Medicine and Hepatology Unit, University of Firenze, Firenze*: Fabio Marra, Valentina Adotti, Martina Rosi, Stefano Gitto.

*Department of Clinical Medicine and Surgery, Hepato‐Gastroenterology Unit, University of Napoli “Federico II”, Napoli*: Pietro Coccoli, Antonio Malerba.

*Department of Clinical Medicine and Surgery, Gastroenterology Unit, University of Napoli “Federico II”, Napoli*: Filomena Morisco, Valentina Cossiga, Mario Capasso.

*Department of Clinical and Experimental Medicine, Hepatology and Liver Physiopathology Laboratory, University Hospital of Pisa, Pisa*: Filippo Oliveri, Gabriele Ricco, Veronica Romagnoli.

The study has been supported, also contributing to the publication, by the Department of Surgery, Oncology and Gastroenterology of the University of Padova (DISCOG) to which the authors are grateful.

## Author Contributions

Conceptualization, FP, SH, BP, and FFa. Methodology, FP, FFa. Software, FT. Formal analysis, FP and FFa. Investigation, FP, SH, BP. Resources, FT. Data curation, BP, AV, EG, VS, GR, MDM, EC, DM, RS, CCe, CCa, AMe, MG, AG, GS-B, FFo, AO, AMa, GN, GR, FA, GV, MB, FT, FFa. Writing—original draft preparation, FP, SH, FFa. Writing—review and editing, BP, AV, EG, VS, GR, MDM, EC, DM, RS, CCe, CCa, AMe, MG, AG, GS-B., FFo, AO, AMa, GN, GR, FA, GV, MB, FT. Visualization, FFa. Supervision, FFa. Project administration, FP, FFa. All authors contributed to the article and approved the submitted version.

## Conflict of Interest

The authors declare that the research was conducted in the absence of any commercial or financial relationships that could be construed as a potential conflict of interest.

## Publisher’s Note

All claims expressed in this article are solely those of the authors and do not necessarily represent those of their affiliated organizations, or those of the publisher, the editors and the reviewers. Any product that may be evaluated in this article, or claim that may be made by its manufacturer, is not guaranteed or endorsed by the publisher.
