# Supplementary material for: Transarterial Chemoembolization for Hepatocellular Carcinoma in Clinical Practice: Temporal Trends and Survival Outcomes of an Iterative Treatment
Source: Front Oncol. 2022 Jan 31;12:822507. doi: 10.3389/fonc.2022.822507 (PMC8841805; doi:10.3389/fonc.2022.822507)
Supplement: Supplementary file 1 [file DataSheet_1.docx]

Supplementary Material

**Supplementary Table 1**. Multinomial logistic regression showing independent factors associated with probability of receive TACE compared to potentially curative treatments (LT, LR and ABL) and palliative therapies (SOR and BSC) in BCLC B patients.

| Variable | | Curative treatment (LT, LR and ABL) | | Palliative treatment (SOR and BSC) | | Curative treatment (LT, LR and ABL) | | Palliative treatment (SOR and BSC) | |
| --- | --- | --- | --- | --- | --- | --- | --- | --- | --- |
|  |  | First treatment | | | | Main treatment | | | |
|  |  | aOR (95% CI) | p | aOR (95% CI) | p | aOR (95% CI) | p | aOR (95% CI) | p |
| Sex | Females  Males | Ref  1.17 (0.69-1.99) | -  0.56 | Ref  0.87 (0.44-1.74) | -  0.69 | Ref  1.07 (0.64-1.80) | -  0.80 | Ref  0.84 (0.42-1.70) | -  0.63 |
| Age (per 10-year increase) | | 0.78 (0.65-0.93) | 0.005 | 1.05 (0.81-1.35) | 0.73 | 0.73 (0.61-0.88) | 0.001 | 1.03 (0.79-1.34) | 0.83 |
| Period of diagnosis | P1  P2  P3  P4  P5  P6 | Ref  0.28 (0.06-1.35)  3.16 (0.98-10.18)  2.37 (0.77-7.29)  1.98 (0.66-5.91)  1.82 (0.60-5.53) | -  0.11  0.05  0.13  0.22  0.29 | Ref  1.06 (0.23-4.77)  3.57 (0.89-14.31)  1.02 (0.25-4.23)  1.26 (0.33-4.82)  0.76 (0.19-3.04) | -  0.94  0.07  0.97  0.73  0.70 | Ref  0.26 (0.05-1.26)  3.83 (1.19-12.30)  3.48 (1.14-10.67)  3.06 (1.03-9.11)  3.11 (1.03-9.36) | -  0.09  0.02  0.03  0.04  0.04 | Ref  1.03 (0.23-4.64)  3.89 (0.97-15.67)  1.21 (0.29-5.01)  1.42 (0.37-5.44)  0.92 (0.23-3.70) | -  0.97  0.06  0.80  0.61  0.91 |
| Surveillance | No  Yes | Ref  1.39 (0.95-2.03) | -  0.09 | Ref  0.72 (0.43-1.20) | -  0.21 | Ref  1.14 (0.78-1.65) | -  0.50 | Ref  0.65 (0.38-1.10) | -  0.11 |
| CRPH | No  Yes | Ref  0.44 (0.30-0.66) | -  <0.0001 | Ref  0.89 (0.51-1.56) | -  0.68 | Ref  0.57 (0.39-0.84) | -  0.004 | Ref  0.92 (0.52-1.63) | -  0.77 |
| MELD | | 0.94 (0.88-1.02) | 0.12 | 1.10 (1.01-1.20) | 0.04 | 0.91 (0.85-0.98) | 0.01 | 1.08 (0.98-1.18) | 0.11 |
| Number | | 0.87 (0.76-0.99) | 0.04 | 1.09 (0.99-1.19) | 0.07 | 0.88 (0.78-0.99) | 0.04 | 1.08 (0.99-1.18) | 0.09 |
| Diameter (cm) | | 0.95 (0.86-1.05) | 0.29 | 1.13 (1.03-1.23) | 0.01 | 0.91 (0.83-0.99) | 0.04 | 1.10 (1.01-1.21) | 0.03 |
| AFP (ng/mL) | ≤20  20-200  >200 | Ref  1.01 (0.63-1.62)  0.82 (0.53-1.26) | -  0.96  0.36 | Ref  0.73 (0.36-1.48)  1.19 (0.67-2.10) | -  0.38  0.56 | Ref  0.89 (0.56-1.42)  0.81 (0.53-1.23) | -  0.63  0.32 | Ref  0.72 (0.35-1.48)  1.22 (0.68-2.18) | -  0.37  0.51 |

TACE treatment is the reference category of the multinomial logistic regression. OR<1 indicates that the variable is associated with higher probability of being treated with TACE rather than the comparison category (curative treatments or palliative treatments). OR>1 indicates that the variable is associated with higher probability to be treated with potentially curative treatments (or palliative treatments) rather than TACE.

Abbreviations: LT, liver transplantation; LR, liver resection; ABL, ablation; SOR, systemic therapy; BSC, best supportive care; aOR, adjusted odds ratio; CI, confidence interval; CSPH, clinically relevant portal hypertension; MELD, Model for End-Stage Liver Disease; MVI, macrovascular invasion; EHS, extrahepatic spread; AFP, alpha-fetoprotein.

**Supplementary Table 2**. Survival analysis according to the period of diagnosis in BCLC B patients.

| Period of diagnosis | Median OS (months) | 5-year survival (%) | aHR (95% CI) ^a^ | p |
| --- | --- | --- | --- | --- |
| All patients | | | | |
| P1  P2  P3  P4  P5  P6 | 16.0 (12.2-19.8)  25.0 (20.3-29.7)  30.0 (24.0-36.0)  35.0 (29.1-40.9)  35.0 (30.0-40.0)  NE (NE-NE) | 15.4  13.6  23.7  26.5  33.8  51.5 | Ref  0.75 (0.53-1.07)  0.75 (0.55-1.03)  0.60 (0.44-0.81)  0.54 (0.40-0.74)  0.40 (0.28-0.57) | -  0.12  0.07  0.001  0.0001  <0.0001 |
| Patients treated with TACE as first therapy | | | | |
| P1  P2  P3  P4  P5  P6 | 16.0 (11.2-20.8)  25.0 (21.8-28.2)  34.0 (25.8-42.2)  34.0 (27.6-40.4)  36.0 (30.7-41.3)  NE (NE-NE) | 6.6  8.2  23.7  25.9  33.5  50.2 | Ref  0.85 (0.56-1.30)  0.60 (0.39-0.90)  0.53 (0.36-0.79)  0.52 (0.35-0.77)  0.38 (0.24-0.61) | -  0.46  0.02  0.002  0.001  <0.0001 |
| Patients treated with TACE as main therapy | | | | |
| P1  P2  P3  P4  P5  P6 | 15.0 (10.9-19.1)  25.0 (21.9-28.1)  30.0 (18.6-41.4)  29.0 (23.8-34.2)  31.0 (22.7-39.3)  47.4 (NE-NE) | 9.3  6.2  17.5  13.4  23.9  53.8 | Ref  0.84 (0.55-1.29)  0.66 (0.44-1.01)  0.60 (0.40-0.91)  0.65 (0.43-0.98)  0.39 (0.24-0.65) | -  0.44  0.06  0.02  0.04  0.0003 |

a) Adjusted for: age, etiology, surveillance, CRPH, MELD, AFP level and main treatment (this latter only in the group including all patients).

Abbreviations: OS, overall survival; aHR, adjusted hazard ratio; NE, not estimable; TACE, trans-arterial chemoembolization.

**Supplementary Table 3**. Univariate and multivariate Cox regression analysis in BCLC B patients.

| Variable | Univariate | | Multivariate | |
| --- | --- | --- | --- | --- |
|  | HR (95% CI) | p | aHR (95% CI) | p |
| Period | | | | |
| P1  P2  P3  P4  P5  P6 | Ref  0.86 (0.63-1.17)  0.76 (0.58-1.00)  0.60 (0.46-0.79)  0.52 (0.40-0.68)  0.41 (0.30-0.56) | -  0.34  0.05  0.0003  <0.0001  <0.0001 | Ref  0.75 (0.53-1.07)  0.75 (0.55-1.03)  0.60 (0.44-0.81)  0.54 (0.40-0.74)  0.40 (0.28-0.57) | -  0.12  0.07  0.001  0.0001  <0.0001 |
| Sex | | | | |
| Female  Male | Ref  0.87 (0.73-1.05) | -  0.14 | - | - |
| Age – 10 years increase | 1.06 (1.00-1.13) | 0.05 | 1.03 (0.95-1.12) | 0.48 |
| Surveillance | | | | |
| No  Yes | Ref  0.81 (0.71-0.94) | -  0.005 | Ref  0.83 (0.71-0.98) | -  0.03 |
| Etiology | | | | |
| Viral  Not viral  Viral + other | Ref  0.83 (0.71-0.97)  1.12 (0.91-1.38) | -  0.02  0.30 | Ref  0.95 (0.78-1.15)  1.15 (0.90-1.48) | -  0.59  0.25 |
| CRPH | | | | |
| No  Yes | Ref  1.42 (1.23-1.65) | -  <0.0001 | Ref  1.21 (1.02-1.45) | -  0.03 |
| Number | 1.02 (1.00-1.04) | 0.09 | -^a^ | - ^a^ |
| Diameter (cm) | 1.02 (1.00-1.04) | 0.09 | - ^a^ | - ^a^ |
| Child-Pugh | | | | |
| A  B7  B8-9 | Ref  1.56 (1.29-1.90)  2.24 (1.83-2.73) | -  <0.0001  <0.0001 | -^b^ | - ^b^ |
| MELD | 1.06 (1.04-1.08) | <0.0001 | 1.04 (1.01-1.07) | 0.006 |
| AFP (ng/mL) | | | | |
| ≤20  20-200  >200 | Ref  1.03 (0.87-1.23)  1.27 (1.08-1.49) | -  0.72  0.003 | Ref  1.00 (0.81-1.23)  1.35 (1.12-1.63) | -  0.99  0.002 |
| Main treatment | | | | |
| BSC  LT  LR  ABL  TACE  SOR  Other | Ref  0.07 (0.04-0.12)  0.18 (0.12-0.26)  0.23 (0.16-0.33)  0.35 (0.25-0.50)  0.46 (0.30-0.70)  0.57 (0.38-0.87) | -  <0.0001  <0.0001  <0.0001  <0.0001  0.0003  0.009 | Ref  0.06 (0.03-0.11)  0.17 (0.10-0.26)  0.23 (0.15-0.34)  0.31 (0.22-0.46)  0.52 (0.32-0.86)  0.46 (0.28-0.75) | -  <0.0001  <0.0001  <0.0001  <0.0001  0.01  0.002 |

a) Not included in multivariate analysis to avoid collinearity with stage.

b) Not included in multivariate analysis to avoid collinearity with MELD

Abbreviations: HR. hazard ratio; CI, confidence interval; aHR, adjusted hazard ratio; CRPH, clinically relevant portal hypertension; MELD, Model for End-stage Liver Disease; AFP, alpha-fetoprotein; BSC, best supportive care; LT, liver transplantation; LR, liver resection; ABL, ablation; TACE, transarterial chemoembolization; SOR, systemic therapies.


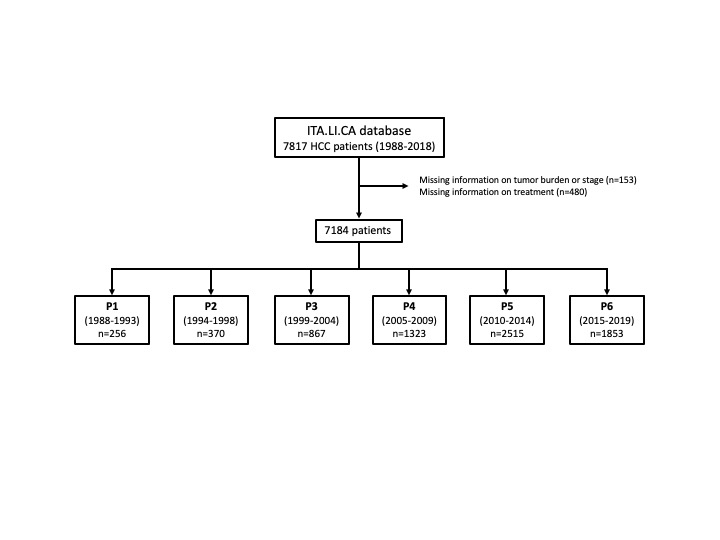


**Supplementary Figure 1**. Flow chart of patient selection.


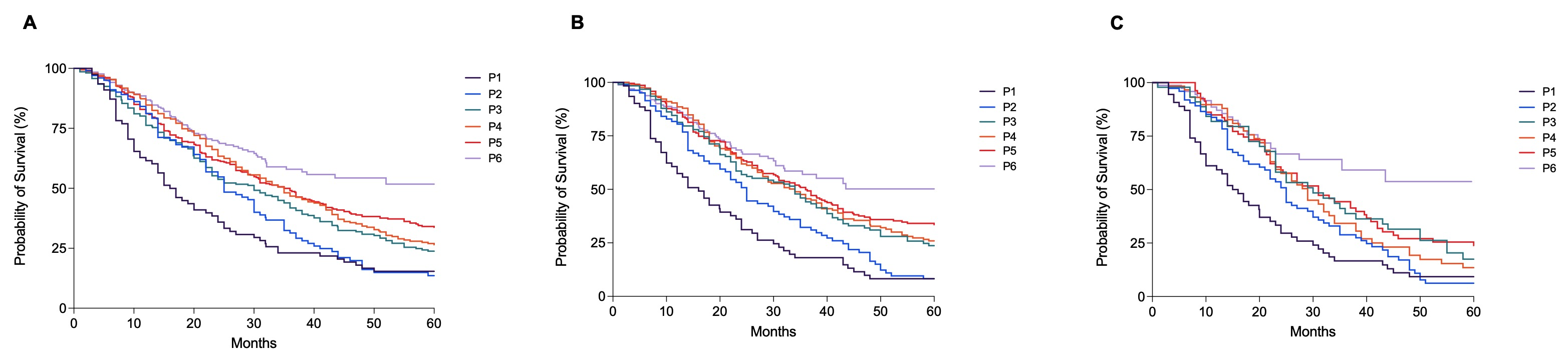


**Supplementary Figure 2**. Kaplan-Meier curves showing overall survival according to the period of diagnosis in BCLC B patients (A), in BCLC B patients treated with TACE as first treatment (B) and in those treated with TACE as main treatment (C) (all p<0.0001).


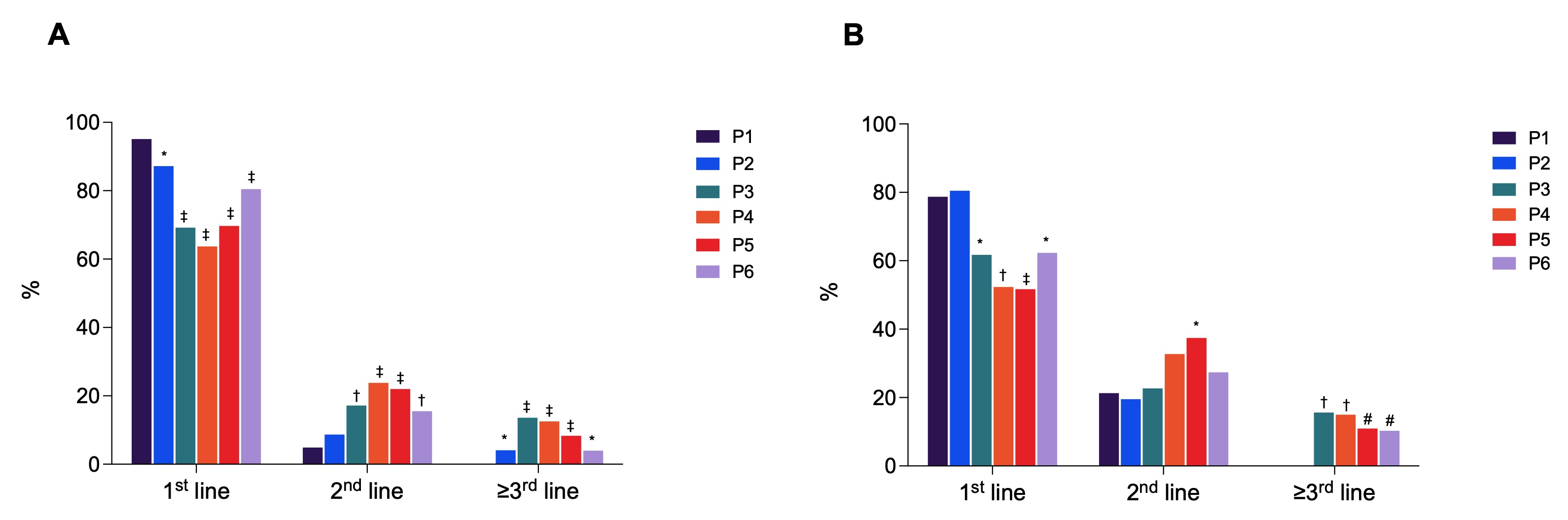


**Supplementary Figure 3**. Proportion of patients treated with TACE overall (A) and in BCLC B stage (B) in 1^st^, 2^nd^ and ≥3^rd^ line divided according to the period of diagnosis (* p<0.05 and ≥0.01; # p<0.01 and ≥0.001; † p<0.001 and ≥0.0001; ‡ p<0.0001).


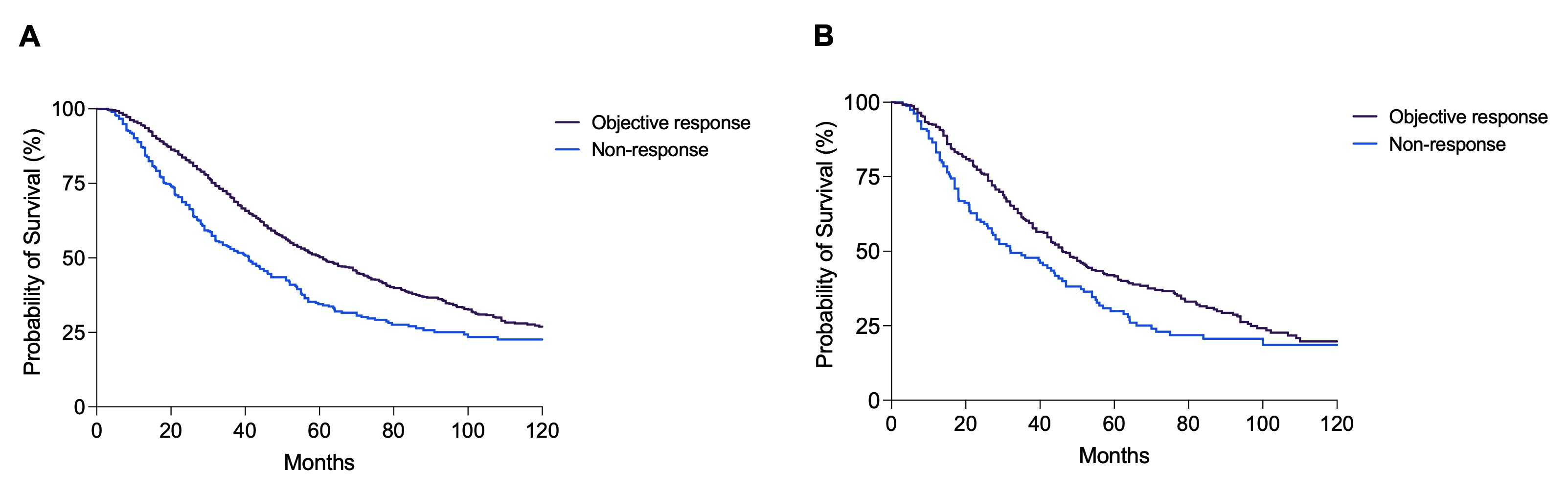


**Supplementary Figure 4**. Kaplan-Meier curves showing overall survival according to the response to the first TACE in the whole patient population (A) and in BCLC B patients (B). Patients with objective response demonstrated a statistically significant longer survival compared to non-responders (p<0.0001 in the whole population and p=0.004 in BCLC B).
